# Lapine periodontal ligament stem cells for musculoskeletal research in preclinical animal trials

**DOI:** 10.1186/s12967-018-1551-2

**Published:** 2018-06-22

**Authors:** H. Chopra, C. Liao, C. F. Zhang, E. H. N. Pow

**Affiliations:** 1 Discipline of Prosthodontics, Faculty of Dentistry, The University of Hong Kong, Hong Kong, SAR China; 2 Discipline of Orthodontics, Faculty of Dentistry, The University of Hong Kong, Hong Kong, SAR China; 3 Discipline of Endodontology, Faculty of Dentistry, The University of Hong Kong, Hong Kong, SAR China; 4 3/F, The Prince Philip Dental Hospital, 34 Hospital Road, Sai Ying Pun, Hong Kong, China

**Keywords:** Periodontal ligament, Stem cells, Surface antigens, Cell differentiation, STRO-1, CD146

## Abstract

**Background:**

Human periodontal ligament stem cells (hPDLSCs) have been shown to be a reliable source of mesenchymal stem cells (MSCs). On the other hand, rabbits have been commonly used in preclinical trials for musculoskeletal research. However, there is a lack of sufficient data on using rabbit periodontal ligament stem cells (rPDLSCs) for regenerative dentistry. This study, for the first time, comprehensively compared rPDLSCs against hPDLSCs in terms of clonogenicity, growth potential, multi-differential capacity and surface antigens.

**Methods:**

Periodontal ligament (PDL) was obtained from the rabbit and human teeth. rPDL and hPDL cells were isolated from PDL using enzymatic digestion method. After culturing for 2 weeks, the cells were first analyzed microscopically. STRO-1^+^CD146^+^ PDLSCs were then sorted from PDL cells by fluorescence-activated cell sorting (FACS) followed by examination of CD34, CD45, CD90, vimentin and desmin markers. The cells were also evaluated by immunohistocytochemical and multi-differentiation potential tests. The clonogenicity and growth of PDL cells were analyzed by Independent *T* test and 2-way repeated measures ANOVA respectively.

**Results:**

rPDL cells were broader and less elongated as compared to hPDL cells. STRO-1^+^CD146^+^ hPDLSCs were isolated from hPDL cells but not from the rPDL cells. Therefore, heterogeneous population of rabbit and human PDL cells were subsequently used for latter comparative studies. FACS analysis and immunohistocytochemistry revealed that rPDL cells were partially positive for STRO-1 as compared to hPDL cells. Furthermore, both rPDL cells and hPDL cells were positive for CD146, CD90, vimentin, and desmin, while negative for CD34 and CD45. No difference in clonogenicity between rPDL and hPDL cells was found (p > 0.05). The proliferative potential of rPDL cells displayed significantly slower growth as compared to hPDL cells (p < 0.05). Osteogenic, adipogenic, and chondrogenic differentiation potential was comparatively less in rPDL cells than that of hPDL cells, but the neurogenic differential potential was similar.

**Conclusion:**

Although rPDL cells manifested variable differences in expression of stem cell markers and multi-differential potential as compared to hPDL cells, they demonstrated the attributes of stemness. Further studies are also required to validate if the regenerative potential of rPDL cells is similar to rPDLSCs.

## Background

Periodontitis, a bacterial-induced inflammatory disorder of the periodontium characterised by the destruction of PDL, cementum and alveolar bone, is one of the major reasons for tooth loss worldwide [1]. The aim of periodontal therapy is to restore/regenerate the PDL to their original form, function and architecture. PDL is a specialized connective tissue that attaches the tooth root to the surrounding alveolar bone. It is composed of a heterogeneous group of cells such as fibroblasts, osteoblasts, cementoblasts, inflammatory cells, endothelial cells, neural, lymphatic cells and epithelial cell rests of Malassez [2]. Besides maintaining stability and supporting the tooth, it helps in sustaining the homeostasis of the tissues, repairing and regenerating damaged tissue. It is known that PDL has one of the highest metabolic turnover rates in the human body [2].

Conventional methods such as guided tissue regeneration (GTR), bone-grafting procedures, application of various growth factors and enamel matrix derivative (EMD) on root surface/bone have been used for quite a long time and demonstrated encouraging results to regenerate periodontal tissues. However, they do have certain clinical limitations, e.g., in cases where there is established loss of periodontal support with horizontal bone resorption, full regeneration of functional periodontal tissue is still impossible [3, 4]. Periodontal regeneration is always challenging due to the complexity of the structure of periodontium and heterogeneity of cells. Therefore, there is a paradigm shift to explore using stem cell-based regenerative therapies as an alternate treatment modality for periodontal regeneration.

Tissue engineering is a multidisciplinary field which entails the use of stem cells, growth factors and scaffolds, which are collectively referred to as the tissue engineering triad [5]. It has been long hypothesised that PDL might contain progenitor cells that can take part in the regeneration of periodontal tissue [6–8], and PDLSCs have been successfully isolated from human PDL [9]. These PDLSCs are multipotent stem cells with clonogenicity, self-renewal properties, and multilineage differentiation potential just like mesenchymal stem cells (MSCs). A study showed that implanting PDLSCs into the subcutaneous tissue of mice could form cementum/PDL-like tissue [9].

Phenotypically, STRO-1 and CD146 are the two earliest mesenchymal stem cell markers identified [9]. These markers have been explored in various studies [10–13] and forms the basis for sorting pure stem cell population from a heterogeneous cell population. PDLSCs from various species such as sheep [14], rat [15], pig [16] and dog [17], have been isolated and characterised. Although rabbit is one of the most widely used small animal model, there is a lack of information on rabbit PDLSCs for regenerative therapies and such data would be useful for preclinical in vitro and in vivo trials using rabbits.

In the present study, we tested the null hypothesis that rPDL cells are no different from hPDL cells regarding clonogenicity, proliferative potential, multi-differentiation potential and the expression of MSC surface markers in vitro.

## Methods

### Rabbit tooth sampling

The study was approved by the “Committee on the use of live animals in teaching and research” (CULATR: 3558-15) of the University of Hong Kong. Three Healthy New Zealand white male rabbits (≈ 9-month-old and weight ≈ 3 kg) were randomly selected. Under sedation of pentobarbital sodium IV (Nembutal, CEVA, Paris, France, 10 mg/kg of body weight) disease-free maxillary premolars of all the rabbits were extracted. The rabbits were then euthanized with an overdose of the same drug to prevent suffering from pain and distress caused by tooth extraction and inability to eat afterwards. The teeth were carefully washed with 95% ethyl alcohol and put in Hanks balanced salt solution (HBSS, Sigma-Aldrich, MO, USA).

### Human tooth sampling

The human maxillary premolars from three patients which were extracted for orthodontic reasons were used (Institutional Review Board, The University of Hong Kong, IRB: UW13-120) in the present study.

### Isolation and culturing

Six maxillary premolars from rabbit and two maxillary premolars from humans were used at one time, and the experiment was performed in triplicate. The teeth were removed from HBSS, and the periodontal ligament (PDL) was carefully scraped off from the middle third of the root using a scalpel. Enzymatic digestion method was used for the isolation of PDLSCs. Briefly, PDL was subjected to 3 mg/mL of collagenase type I (Gibco; Thermo Fisher Scientific, MA, USA) and 4 mg/mL of dispase II (Gibco) solution for 1 h at 37 °C. These cells were then passed through a 70 µm strainer (BD Biosciences, NJ, USA) to obtain single cell suspensions and were dispersed into T-75 flasks. The flasks were incubated at 37 °C and 5% CO_2_ for 2 weeks in complete culture medium [CCM: alpha modification of Eagle’s medium (α-MEM supplemented with 15% FBS along with 1% penicillin–streptomycin, 2% l-glutamine)] (All from Gibco) with medium change every 3 days. All the subsequent experiments were performed in either P2 or P3 passage. Further, the same passage was used for the same experiment.

### Colony formation unit-fibroblast (CFU-F) assay

CFU-F assay was performed by seeding 200 cells of rPDL cells and hPDL cells at the P2 passage in three 100 mm dish for 10 days. The medium was replaced every 3 days. After 10 days, the cells were fixed using 4% paraformaldehyde (PFA) for 30 min and washed twice with PBS followed by staining with crystal violet. A colony with greater than or equal to 50 cells was considered as a colony otherwise they were not counted. CFU was calculated by dividing the total number of colonies by the initial number of cells and multiplied by 100 as a percentage. The experiment was conducted in triplicate.

### Proliferative potential/growth curve

The proliferative potential of PDL cells was determined by the CCK-8 assay (Sigma-Aldrich). In brief, rPDL cells and hPDL cells at P2 passage were seeded in 96-well plates at a density of 1000 cells/well and cultured in CCM at 37 °C in a humidified atmosphere containing 5% CO_2_. The growth was measured every 2 days for 2 weeks. The medium was replaced every 3 days. On the day of evaluation, CCM was replaced by phenol red-free culture medium containing 5% FBS, 1% P/S (working medium) and 10 µL of CCK-8 solution was added (Sigma-Aldrich), and the plates were incubated for 2 h at 37 °C. Afterwards, the absorbance was measured at 450 nm by a microplate reader. The experiment was performed in triplicate with each set having three wells. The negative control consisted of only working medium and 10 µL of the CCK-8 solution.

The growth values of each experiment group at each time point was calculated by subtracting the mean of negative control values from the values of the experimental group at that time point.

### Multipotent differentiation potential

Both rPDL cells and hPDL cells were tested for osteogenic, neurogenic, adipogenic and chondrogenic differentiation potential. Each experiment was replicated thrice.

#### Osteogenic differentiation

rPDL cells and hPDL cells at P3 were seeded in 6-well plates at a density of 1.5 × 10^4^ cells/well incubated in complete culture medium for 24 h at 37 °C in a humidified atmosphere containing 5% CO_2_. After 24 h, medium in treatment group was changed to customized osteogenic differentiation media containing 85% α-MEM, 10% FBS, 1% penicillin–streptomycin, 2% l-glutamine (Thermo Fisher Scientific, MA, USA), 0.5% (50 µg/mL) l-ascorbic acid-2-phosphate, 0.5% (10 nM) dexamethasone, 1% (10 mM) β-glycerophosphate (All from Sigma-Aldrich). On the other hand, the control group was supplemented with only 87% α-MEM, 10% FBS, 1% penicillin–streptomycin, and 2% l-glutamine. Both the mediums were refreshed at 3-day intervals for 4 weeks. The wells were then evaluated for osteogenic differentiation by histochemical staining (HS) with Alizarin Red S (ARS) (p.H.:4.1–4.3; Sigma-Aldrich).

#### Neurogenic differentiation

rPDL cells and hPDL cells at P3 were seeded in two 6-well plates at a density of 1.5 × 10^4^ cells/well incubated in complete culture medium for 24 h at 37 °C in a humidified atmosphere containing 5% CO_2_. After 24 h, medium in treatment group was changed to customized neurogenic differentiation media containing Neurobasal-A medium supplemented with 2% B-27 (Both from Gibco-Lifesciences) with 20 ng/mL epidermal growth factor, 40 ng/mL fibroblast growth factor (Both from BD Biosciences, San Jose, CA, USA) and 1% penicillin–streptomycin. The control group was supplemented with α-MEM and 1% penicillin–streptomycin only. Both the mediums were refreshed at 3-day intervals for 3 weeks. One 6-well plate was analyzed by HS while the other plate was examined for immunohistocytochemistry (IHC).

### Adipogenic differentiation

rPDL cells and hPDL cells at P3 were seeded at a density of 4 × 10^4^ cells in 6-well plates and incubated at 37 °C, in a humidified atmosphere of 5% CO_2_. The complete culture medium was replaced every 2–3 days until the cells reaches 100% confluence (3–4 days). At near 100% confluence, α-MEM was removed, and 3 cycles of adipogenic induction media (AIM) and adipogenic maintenance media (AMM) were initiated in treatment groups while the control group was supplemented with AMM only. The AIM and AMM (Adipogenic differentiation bullet kit: Lonza, Walkersville, MD, USA) were prepared as per the manufacturer directions [AIM-Adipogenic induction medium, h-insulin (recombinant), l-glutamine, Mesenchymal cell growth supplement (MCGS), Dexamethasone, Indomethacin, IBMX (3-isobuty-l-methyl-xanthine) and GA-1000; AMM-Adipogenic maintenance medium, h-insulin (recombinant), l-glutamine, MCGS and GA-1000]. After 3 cycles, both the groups were maintained in AMM for 7 days with the replacement of AMM twice at an interval of 3 days. The wells were then analyzed for adipogenic differentiation by HS with Oil Red O (ORO) (Sigma-Aldrich).

#### Chondrogenic differentiation

Chondrogenic assays were performed in accordance with the manufacturer’s protocol (Lonza). Briefly, 3 × 10^6^ PDL cells from both human and rabbit were aliquoted to two equal groups, treatment group and the control group in polypropylene tubes. In the treatment group, 1.5 × 10^6^ cells were washed with incomplete chondrogenic medium (Lonza) and centrifuged at 1200 rpm for 5 min at room temperature (RT). These cells were resuspended in complete chondrogenic medium. It is imperative to note that 1 µL of TGF-β3 (Sigma-Aldrich) will convert 2 mL of incomplete chondrogenic medium to complete chondrogenic medium. Three tubes containing cells at a concentration of 0.5 × 10^6^ from both the treatment and control group were centrifuged at 1200 rpm for 5 min at RT. The caps were loosened without disturbing the pellets and kept for incubation for 4 weeks with medium refreshed every 3 days. The cell pellets were then evaluated for chondrogenic differentiation by HS with H & E staining.

### Histochemical staining (HS)

For the osteogenic assay, after 4 weeks of culture, mediums of both the treatment and control group were removed. The wells were then washed twice with PBS for 2 min followed by incubation of cells with 4% paraformaldehyde (PFA) for 30 min at RT. After fixation, PFA was removed, and the wells were again washed twice with PBS for 5 min. Fresh prepared ARS was added to all wells and incubated for 30 min at RT. After staining, ARS was removed and washed with PBS until excess stain was no longer apparent. The plates were then scanned with a scanner and images of cells were taken by the phase contrast microscope.

For the neurogenic assay, the same protocol was followed as described above, except that, freshly prepared Cresyl Violet stain (p.H.:3.7–3.9; Sigma-Aldrich) was used.

For the adipogenic assay, working solution of ORO was prepared by mixing 3 parts (30 mL) of ORO stock solution with 2 parts (20 mL) of deionized water followed by filtering with 0.2 µm syringe filter. All the remaining procedures were same as described above except that after fixation, 60% isopropanol (Sigma-Aldrich) was added to each well for 5 min at RT followed by staining with ORO.

For the chondrogenic assay, the pellets were fixed, embedded, sectioned (5 µm) and dewaxed. These pellets were then stained with freshly filtered Hematoxylin (Sigma-Aldrich) for 5 min followed by washing for 3 min and then staining with Eosin stain (Merck, NJ, USA) for 2 s and again washed for 1 min. The specimens were then mounted with coverslips with Permount (Fisher Scientific) and analyzed by the phase contrast microscope.

### Immunohistocytochemistry (IHC)

The cells from another set of neurogenic assays were washed twice with PBS for 2 min and fixed in cold 4% PFA for 30 min. These were then permeabilized with 0.25% Triton-X100 (USB Corp., Cleveland, OH, USA) in PBS for another 30 min followed by blocking in 1% Bovine serum albumin (BSA) (Sigma-Aldrich) for 1 h. All the above procedures were carried at RT. The cells were then incubated with primary mouse antibody, anti β-3 Tubulin (G7121; Promega, WI, USA) at 1:200 dilution for overnight at 4 °C. Subsequently, the cells were incubated with secondary antibody FITC Goat Anti-Mouse IgG/IgM (BD Pharmingen, BD Biosciences, NJ, USA). Cells were then counterstained with DAPI (Sigma-Aldrich) and analyzed with a fluorescent microscope.

IHC was also used for analyzing the expression of CD34, CD45, STRO-1, CD146, CD90, Vimentin and Desmin. The above-described protocol for β-3 Tubulin was also followed except that cells were not permeabilized. The antibodies and their corresponding isotype controls are described in Table 1.

**Table 1:** Antibodies and isotype controls

		React.	Clone	Conj^n^	Flourochrome	Isotype control
CD 34	GTX28158^a^	R, H	MEC 14.7	Unconj.	–	
	46-4817-80^b^	–	r2a-1B2	2° Ab	PerCP eFlour710	46-4724-82^b^
CD45	GTX74646^a^	R, H	MEM	Conj.	FITC	GTX35015^a^
STRO-1	MAB1038^c^	X, H	STRO-1	Unconj.	–	
	555988^d^	–	–	2° Ab	FITC	GTX35040^a^
CD146	48-1469-41^b^	R	P1H12	Conj.	eFluor^®^ 450	48-4714-80^b^
CD90	Ab226^e^	R	MRC 0X7	Conj.	FITC	GTX35015^a^
Desmin	50-9747-82^b^	R, H	DE-U-10	Conj.	eFluor^®^ 660	50-4714-82^b^
Vimentin	GTX79851^a^	R, H	VI-RE/1	Conj.	PE	GTX35017^a^

*R* Rabbit, *H* Human, *X* Not present for Rabbit

^a^ Genetex, ^b^ Ebioscience, ^c^ R&D, ^d^ BD Biosciences, ^e^ Abcam

### Flow cytometry

Fluorescence-activated cell sorting (FACS) was used for quantitative analysis of the phenotype of the cells. Both rPDL cells and hPDL cells were used in a concentration of 0.1 × 10^6^ to 0.5 × 10^6^ cells. The cells were simultaneously blocked (0.1% BSA) and incubated with the antibody (Table 1) for 30–45 min in an ice box with gentle stirring. If the secondary conjugated antibody was used, the sample was further incubated with it for further 30–45 min after washing twice with cold PBS for 5 min. After incubation with either the conjugated or secondary antibody, the samples were again washed thrice with PBS. The negative control consisted of unstained cells whereas isotype control had cells with isotype of the corresponding antibody and incubated for 30–45 min followed by washing thrice for 5 min in PBS. All the samples were strained with 70 µm filter to obtain singlets and then subjected to BD LSR Fortessa™ (BD Biosciences) for analysis of respective markers. Minimum 20,000 events were recorded. The data were analyzed by FlowJo Version 10.0 (FlowJo, LLC, Ashland, OR, USA).

### Statistical Analysis

Difference in the mean CFU percentage between groups was analyzed by Independent T-test. Regarding the growth curve, 2-way repeated measures ANOVA was applied for the testing difference in mean growth between two groups (rPDL cells and hPDL cells) at the same time point and between different time points within the same group. The pairwise comparisons were adjusted by Bonferroni adjustment. The above tests were performed as the two-sided tests at the 0.05 significance level using IBM SPSS Statistics 24 (IBM Corp. Armonk, NY, USA).

## Results

### Teeth

**Fig. 1:**
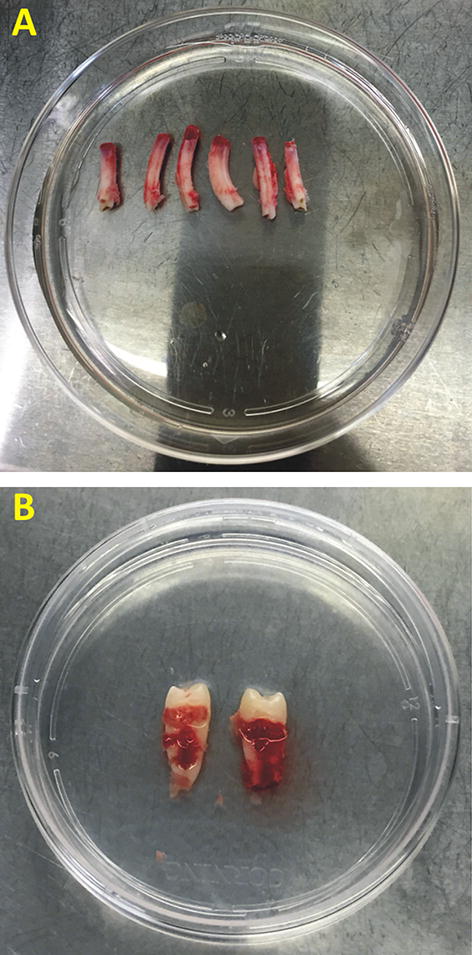
Extracted teeth with PDL in Hanks balanced salt solution (HBSS) **A** Rabbit **B** Human

The extracted rabbit teeth were largely cylindrical with an open apex (Elodont dentition-teeth that grow throughout life) in comparison to human premolars which had a constriction between crown and root and a closed apex (Fig. 1).

### Isolated rPDL cells and hPDL cells

**Fig. 2:**
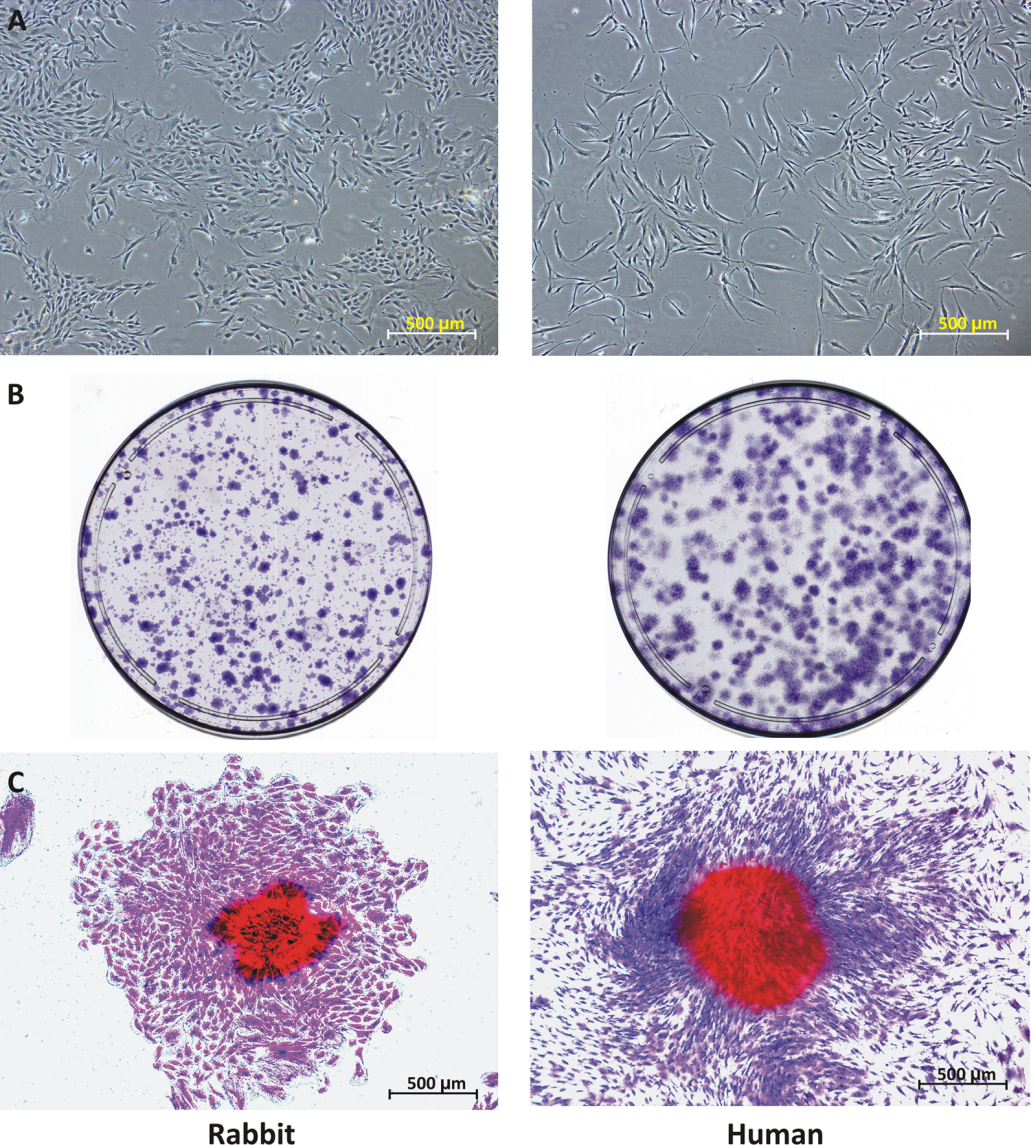
**A** Morphology of rPDL cells and hPDL cells after 2 weeks of culture [4X (0.52 µm/px)]. **B** CFU-assay after staining rPDL cells and hPDL cells with crystal violet at day 10 in 100 mm dish. **C** The magnified colony with greater than 50 cells [4X (0.52 µm/px)]

The cells from digested PDL of rabbit teeth and human teeth reached confluency in approximately 2 weeks. It was observed that rPDL cells were broader in size but less elongated as compared to hPDL cells (Fig. 2A).

### CFU assay

The mean CFU % of rPDL cells and hPDL cells was 1.31 (S.D. = 0.07) and 1.41 (S.D. = 0.15) respectively, and no statistically significant difference was found between two groups (p = 0.33) (Fig. 2B, C).

### Growth curve

**Fig. 3:**
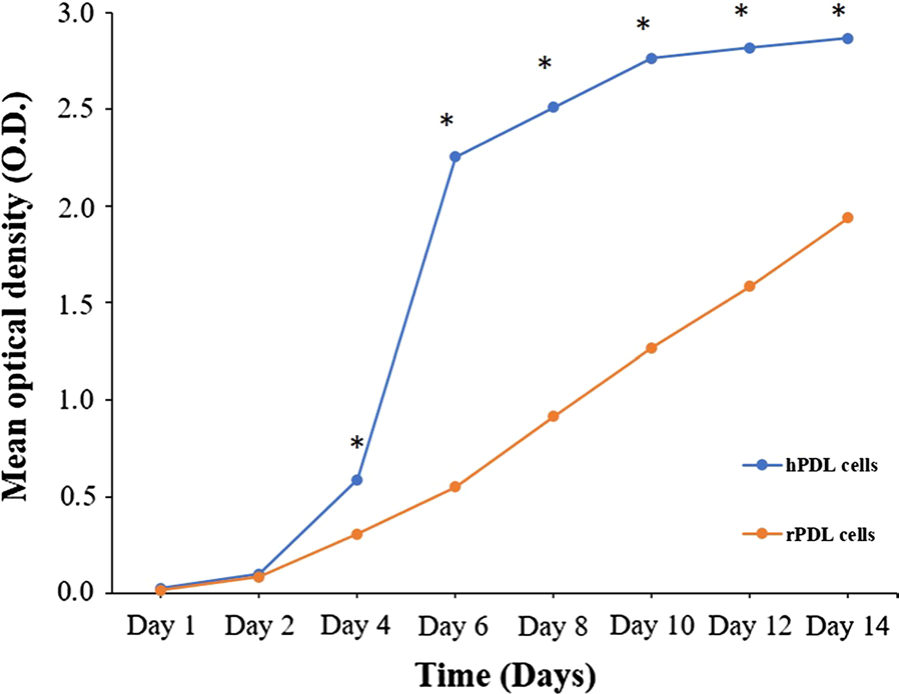
Growth curves of rPDL cells (orange) and hPDL cells (blue) observed over a period of 2 weeks

The growth data are displayed in Table 2 which showed a statistically significant difference in overall time points (p < 0.05) as wells as between time points (p < 0.05) and groups (p < 0.05). Significant growth was observed between all the time points in both cells from day 1 to day 8 (p < 0.05). rPDL cells grew continually from day 10 to day 14 (p < 0.05) while no significant growth was found in the hPDL cells (p > 0.05). Overall, rPDL cells were proliferating significantly slower than hPDL cells (p < 0.05), except on day 1 and day 2 (Fig. 3).

**Table 2:** Growth curve of rPDL cells and hPDL cells by plotting mean optical density over time

Days	Groups^a^	Mean ± standard deviation (N = 9)	Pairwise comparison^b^		
			Between groups	Within group	
			P value	Group 1	Group 2
Day 1	1	0.017 ± 0.007	0.182	Day 1 <	Day 1 <
	2	0.025 ± 0.016		Day 2 <	Day 2 <
Day 2	1	0.085 ± 0.019	0.437	Day 4 <	Day 4 <
	2	0.100 ± 0.054		Day 6 <	Day 6 <
Day 4	1	0.306 ± 0.061	0.000*	Day 8 <	Day 8 <
	2	0.588 ± 0.061		Day 10 <	Day 10 =
Day 6	1	0.551 ± 0.099	0.000*	Day 12 <	Day 12 =
	2	2.255 ± 0.148		Day 14	Day 14
Day 8	1	0.915 ± 0.186	0.000*		
	2	2.510 ± 0.144			
Day 10	1	1.268 ± 0.189	0.000*		
	2	2.764 ± 0.075			
Day 12	1	1.585 ± 0.070	0.000*		
	2	2.818 ± 0.052			
Day 14	1	1.939 ± 0.121	0.000*		
	2	2.868 ± 0.060			

*S.E* standard error

^a^Group 1: rPDL cells; Group 2: hPDL cells

^b^Adjustment for multiple comparisons: Bonferroni

* Statistically significant at p < 0.05

### Multipotent differentiation potential

#### Osteogenic differentiation

**Fig. 4:**
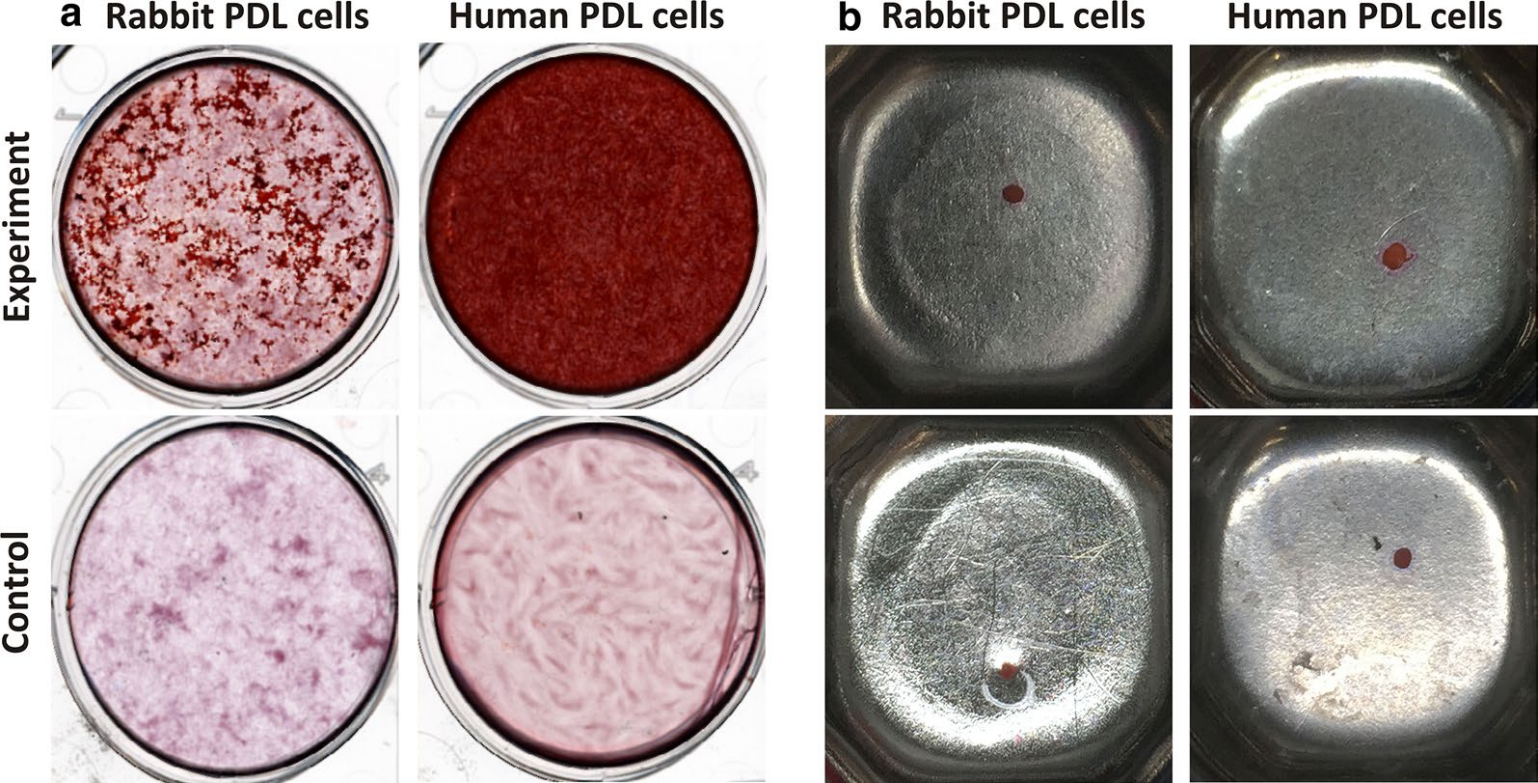
Multi-differentiation potential of PDL cells (visual examination) **a** PDL cells in osteogenic differentiation media for 4 weeks. **b** PDL cells in chondrogenic differentiation media for 4 weeks as pellets

**Fig. 5:**
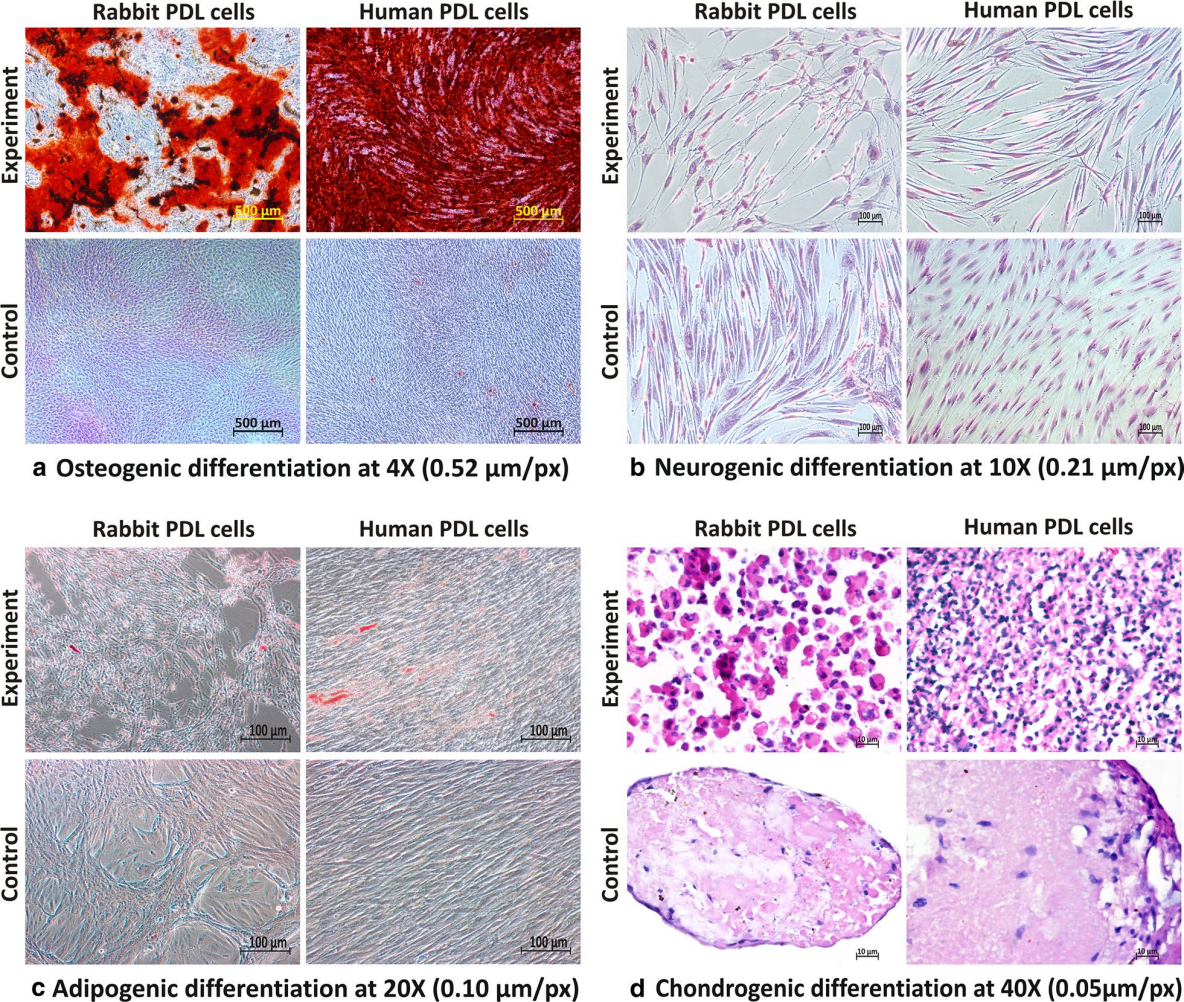
Multi-differentiation potential of PDL cells (microscopic examination) **a** PDL cells were grown in osteogenic differentiation media for 4 weeks and then stained with ARS. Red color represented mineralized nodules after PDL cells differentiated to osteoblast like cells. **b** PDL cells were grown in neurogenic differentiation media for 3 weeks and then stained with Cresyl violet stain. Change in morphology represented differentiation of PDL cells to neuronal like cells. **c** PDL cells were grown in AIM and AMM and stained with ORO. Large number of orange lipid vacuoles were seen in both rPDL cells and hPDL cells. **d** PDL cells were grown in chondrogenic differentiation media for 4 weeks. H & E staining revealed dark blue differentiated nuclei representing chondrocyte like cells in both rPDL cells and hPDL cells as compared to controls

Both rPDL cells as well as hPDL cells differentiated into osteoblast-like cells under osteogenic conditions producing calcific nodules that appeared red on staining with ARS. Visual examination suggested that hPDL cells produced more mineralised nodules than rPDL cells, with wells attaining deep red as compared to rPDL cells where wells were light red (Fig. 4a). Further, the above findings were confirmed by microscopic examination (Fig. 5a).

#### Neurogenic differentiation

**Fig. 6:**
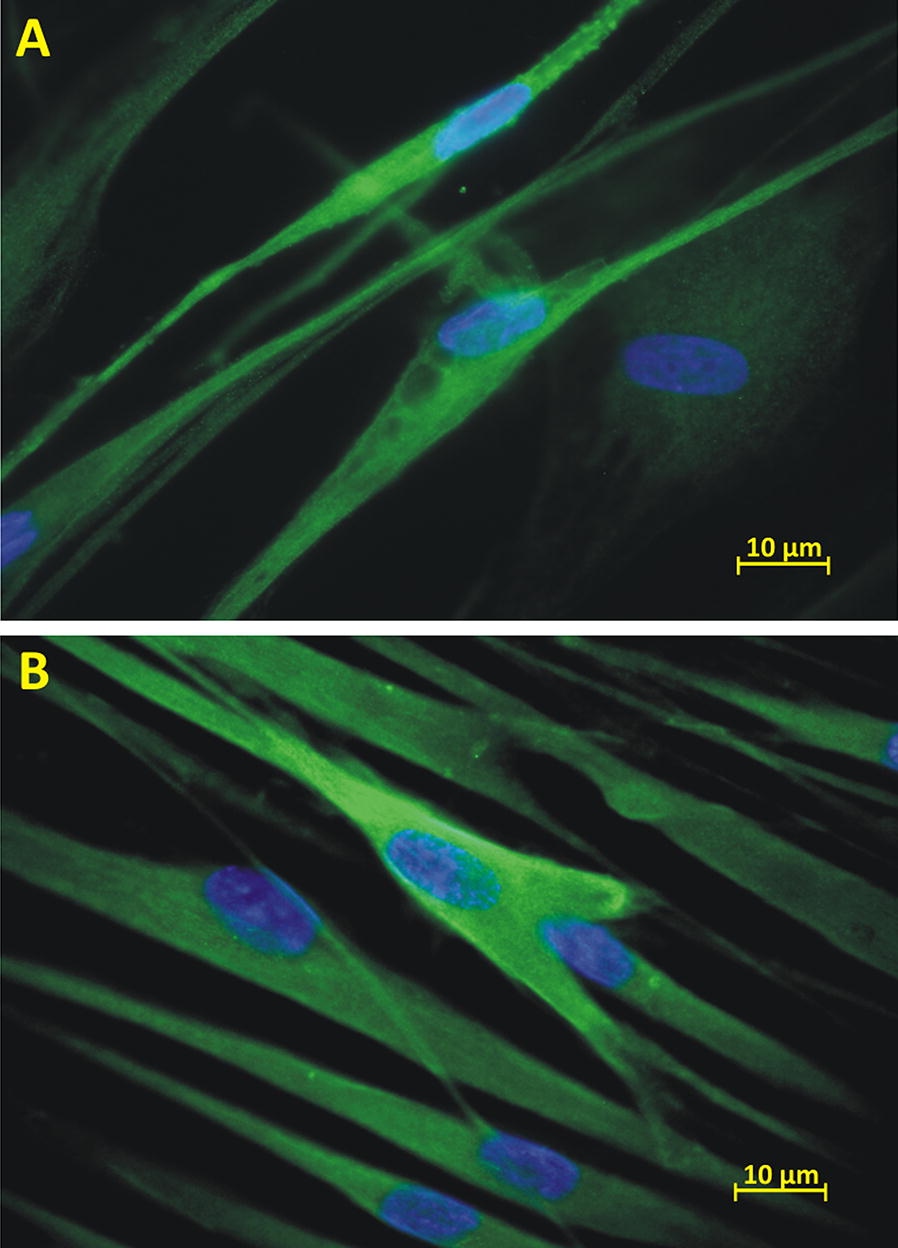
Immunohistocytochemical analysis for Neurogenic assay. Immunostaining of PDL cells with Anti β-3 tubulin shows characteristic neuron like fluorescence in both **A** Rabbit PDL Cells **B** Human PDL Cells. Magnification: 100X (0.24 µm/px)

Both rPDL cells, as well as hPDL cells, showed a characteristic change in morphological appearance after incubating with neurogenic induction medium (NIM) as compared to the control (Fig. 5b). Further, the effect of NIM was more noticeable in rPDL cells as compared to hPDL cells (Fig. 5b). Additionally, on immunofluorescence staining with anti β-3 Tubulin, both cell populations showed characteristic fluorescence (Fig. 6).

#### Adipogenic differentiation

After adipogenic induction, both rPDL cells, as well as hPDL cells, manifested abundant orange lipid vacuoles on staining with ORO (Fig. 5c). It was further observed that mature lipid vacuoles (dark red) were more pronounced in hPDL cells as compared to that of rPDL cells.

#### Chondrogenic differentiation

During 4-week period, the pellets were firstly observed regarding compactness. With the same number of cells, it was found that pellets of rPDL cells were not dense as compared to hPDL cells (Fig. 4b). This was further confirmed during processing that rPDL cells disintegrated very easily as compared to hPDL cells.

H & E staining revealed that hPDL cells stained intensely particularly the nuclei as compared to rPDL cells confirming the differentiation of PDLSCs into chondrocytes like cells (Fig. 5d).

### Immunohistocytochemistry

**Fig. 7:**
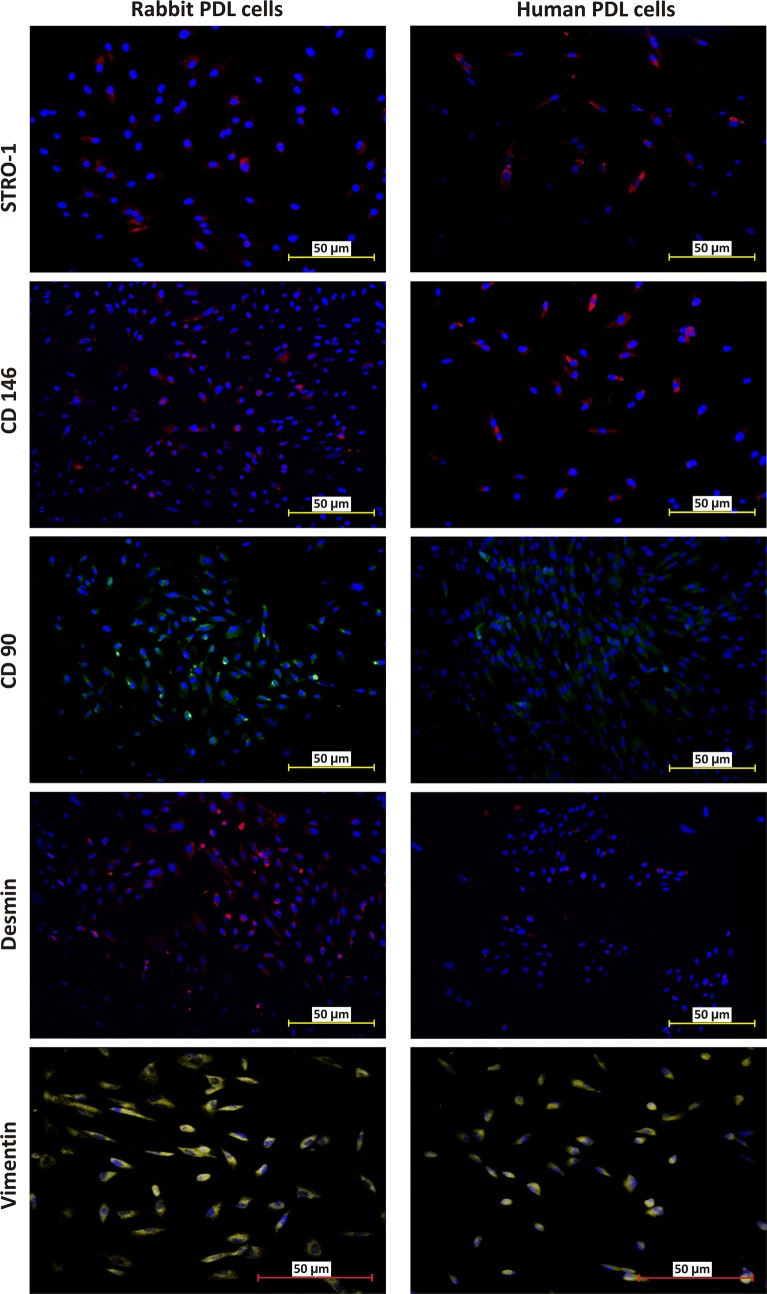
Immunohistocytochemical analysis of rPDL cells and hPDL cells. rPDL cells were partially positive for STRO-1 as compared to hPDL cells. Both rPDL cells and hPDL cells were positive for CD146, CD90, Vimentin and Desmin. CD34 and CD45 were negative in both rPDL cells and hPDL cells (data not shown). Magnification: 20X (0.44 µm/px)

Immunohistocytochemical results showed that both rPDL and hPDL cells were positive for STRO-1, CD146, CD90, Vimentin and Desmin but negative for CD34 and CD45 (Fig. 7). However, rPDL cells appeared to be less positive to STRO-1 as compared to hPDL cells.

### Flow cytometry

**Fig. 8:**
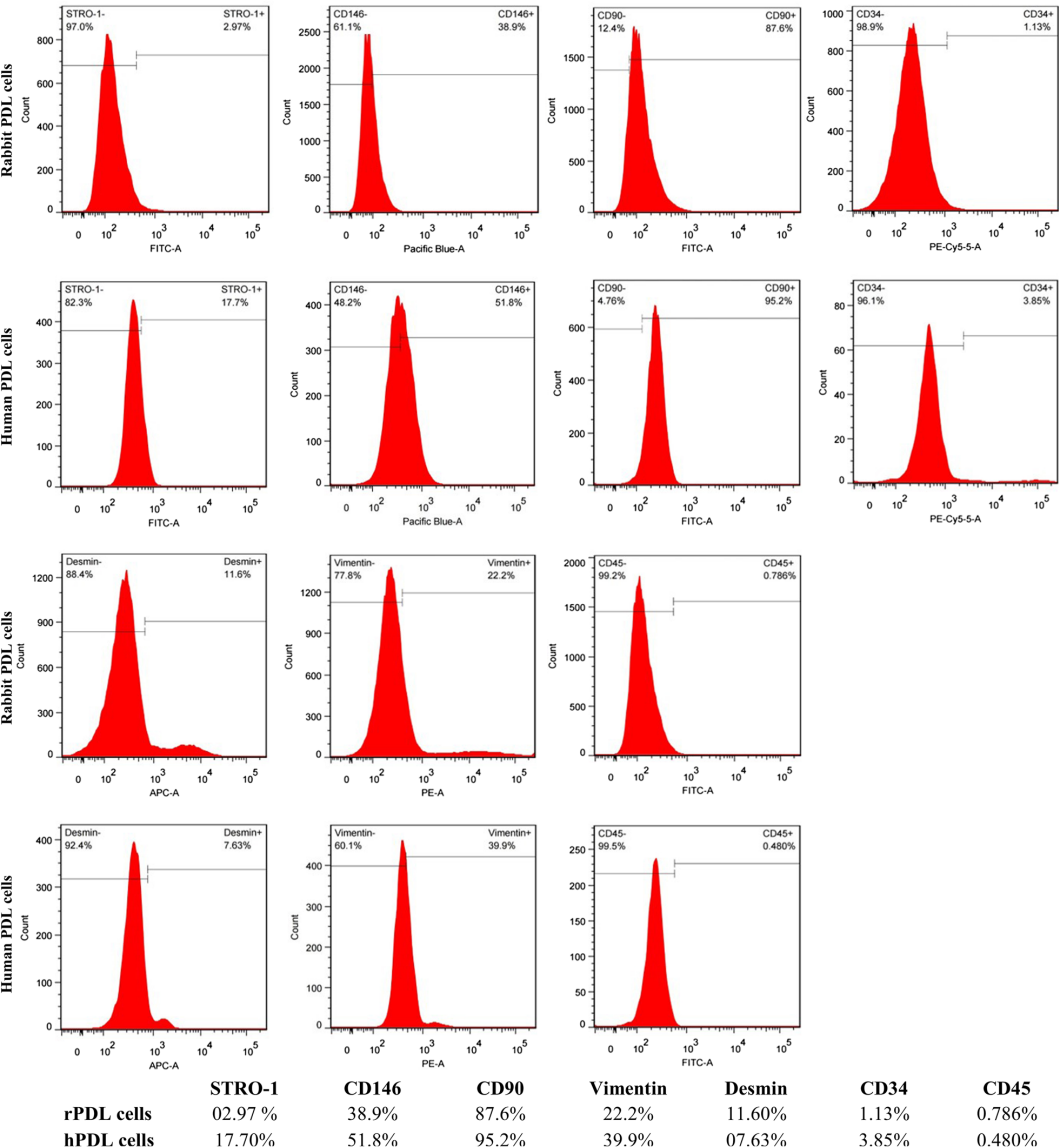
Flow cytometric analysis. The quantitative expression of stem cell surface markers on rPDL cells and hPDL cells

FACS analysis revealed almost similar expression for both rPDL cells and hPDL cells except for STRO-1 (Fig. 8). rPDL cells were partially positive for STRO-1 (2.97%) while hPDL cells were comparatively more positive for STRO-1 (17.70%). Both rPDL cells and hPDL cells were strongly positive for CD90. Expression of other cell surface markers including CD146, Vimentin and Desmin were also found to be positive in both rPDL cells and hPDL cells. However, haematopoietic stem cell markers such as CD34 and CD45 were absent in both cell populations.

## Discussion

Human PDLSCs are well studied, but there is a lack of data concerning rabbit PDLSCs although rabbit has been used as the preclinical animal model in 35% of musculoskeletal research studies [18]. In this study, rPDL cells were isolated, characterised and compared with hPDL cells.

The rabbit or human extracted teeth were transported in freshly prepared sterile HBSS (< 15 days). HBSS was used as a transport media for avulsed tooth because it has been shown to be the optimal medium to maintain PDL cell viability for > 90% [19]. Further, freshly prepared HBSS was used because storage time had a negative influence on PDL cell viability [20]. Regarding temperature, although conflicting results were reported [21, 22], cold storage box was used in the present study to prevent any possible detrimental effect.

The periodontal ligament was scrapped from the middle third of the root because it was substantiated that proliferating mesenchymal cells were present predominantly paravascularly in the middle portion of the root [23]. PDLSCs can be isolated by two methods; outgrowth and enzymatic digestion method [24]. The latter approach was used because it has greater proliferation rate, better CFU-F efficiency and higher differentiation potential than the outgrowth method [25]. Regarding the enzymatic method, Collagen/Dispase combination was used instead of Trypsin/EDTA because it was documented to be 20% more efficient in isolating PDLSCs [26].

The culture medium is essential for defining cell properties. There are two common types of culture medium for MSCs; Dulbecco’s minimum essential medium (DMEM), and alpha modification of Eagle’s medium (α-MEM). In the current study, α-MEM was used because it has been shown that PDSLCs cultured in α-MEM had greater proliferation rates and osteogenic potential as compared to DMEM [27].

STRO-1 and CD146/MUC18 are markers of mesenchymal stem cells [9, 28–30], and they are usually used for isolating hPDLSCs from heterogeneous hPDL cells [10, 11]. In the present study, the same set of markers had been tried for rPDLSCs isolation. However, despite multiple attempts, we failed to isolate STRO-1^+^/CD146^+^ rPDLSCs cells. On the contrary, we were able to isolate STRO-1^+^/CD146^+^ hPDLSCs (data not shown) and the proportion of which is similar to the previous studies [10, 11]. Therefore, to make our study comparable with the human cells, we used the heterogeneous population of both PDL cells in all sets of downstream experiments.

FACS analysis revealed that rPDL cells were less positive to STRO-1 (2.97%) than hPDL cells (17.70%). Similar expression pattern was also found in IHC results which was in agreement with a previous study [12]. The present study was the first to quantitatively analyze the expression of STRO-1 in rPDL cells. No such data have been published because no specific anti-rabbit STRO-1 antibody is currently available and the expression of STRO-1 by rPDL cells, according to our data, seems to be weak. On the contrary, the expression of STRO-1 in hPDL cells has been found to be variable from 4.1 to 27% [28–32] and our results were in line with these studies.

CD146 is a transmembrane glycoprotein, that is also referred to as S-Endo 1–associated antigen [33] or MelCAM/MUC18 [34]. Although it is known as a marker of endothelial cell lineage [35], it can also be found in many other cells such as melanoma cells [34], smooth muscle cells [33], follicular dendritic cells [33] and periodontal ligament stem cells (PDLSCs) [9, 28–30]. Similar to STRO-1, the expression of CD146^+^ in hPDL cells is variable from 7.8 to 88.2% [28, 29, 31, 36]. Despite anti-rabbit CD146^+^ antibody was used, the CD146^+^ expression of hPDL cells (51.8%) was in accordance with the previous studies [28, 29, 31, 36]. rPDL cells (38.9%) had less CD146^+^ as compared to hPDL cells, which was also confirmed by IHC results. Presence of sufficient expression of CD146^+^ in rPDL cells implies that in future studies, CD146^+^ could be considered for isolating homogeneous stem cell population from a heterogeneous rPDL cell population, where amelioration of osteogenic activity is desired [13].

CD90 is a membrane glycoprotein which is also referred to as Thy 1 because it is discovered as a thymocyte antigen [37]. It is the most common stem cell marker. Our results showed that the expression of CD90 in the rPDL cells (87.6%) were comparable with the hPDL cells (95.2%) and similar findings were observed in the IHC results.

Vimentin and Desmin are type III intermediate filament proteins that are encoded by VIM gene and DES gene respectively. Vimentin is of mesenchymal origin whereas desmin is myogenic in origin [38]. Our IHC results demonstrated the presence of vimentin in hPDL cells which is in agreement with the previous similar studies [12, 39, 40]. In addition, the present study was the first to quantitatively analyze vimentin in both rPDL cells and hPDL cells. Vimentin was expressed more in hPDL cells (39.9%) than rPDL cells (22.2%). Desmin, on the other hand, is the major muscle-specific intermediate filament protein. It is present in skeletal, cardiac, and smooth muscles. It helps in maintaining the structural integrity and smooth muscle function [41]. It was evaluated in the present study because it might play a role in maintaining the integrity of vascular tubes after co-culturing with endothelial cells, in our future studies. Similar expression of desmin in both rPDL cells (11.6%) and hPDL cells (7.63%) was found in FACS and IHC analyses. The above results were, however, contrary to a study which found that PDL cells would form myotube and express desmin only after pre-treatment of the cells with 5-aza 2 deoxycytidine [42].

CD34 and CD45 are haematopoietic stem cell markers. Our IHC results revealed the absence of both markers in hPDL and rPDL cells and that conformed to the previous studies [28–30, 42].

Clonogenicity is one of the cardinal properties of stem cells. The CFU-assay is the standardised assay where cells are seeded in low densities to determine their ability to form colonies. The clonogenic potential of rPDL cells as determined by CFU-assay was comparable to hPDL cells. However, the colonies were much smaller in size as compared to hPDL cells.

The proliferative potential of PDL cells was determined by using CCK-8 which is a highly versatile and quantitative assay. Other advantages of CCK-8 include inertness (non-reactivity with the medium), high sensitivity and more stability (degradation at the longer time) than MTT. The smaller size of rPDL colonies can be attributed to the fact that rPDL cells growth was slower than hPDL cells.

Another standard criterion of stem cells is multi-differentiation potential. In the present study, four currently available differentiation assays; osteogenic, neurogenic, adipogenic, and chondrogenic assays were evaluated in both rPDL cells and hPDL cells. Our findings demonstrated that rPDL cells can form mineralized deposits, and lipid vacuoles in vitro, although to a lesser extent as compared to hPDL cells. Further, H&E staining images showed that rPDL cells differentiated into chondrocytes to a much lesser degree as compared to hPDL cells. However, evaluation of neurogenic differentiation by microscopic analysis, as well as IHC displayed similar results in both rPDL cells and hPDL cells. The results from neurogenic differentiation are expected because dental tissue along with other craniofacial connective tissues are formed by a special type of mesenchymal tissue called as ectomesenchyme which in turn is derived from the neural crest during embryonic development [43]. Since the dental stem cells share the common origin with neural tissues, they showed significant neurogenic differentiation in our study.

It should be noted that we used heterogenous rPDL and hPDL cells in all our experiments which is one of the major limitation of the study. However, despite of being heterogenous in nature, our results showed that the PDL cells demonstrated certain properties of MSC cells but to a lesser extent. Therefore, further studies should be done to validate the potential of using rPDL cells instead of rPDLSCs in stem cell research. Otherwise, if the full regenerative potential of homogenous rPDLSCs is to be studied and compared with the human ones, specific markers for rabbits should be developed to increase the yield during the isolation process.

## Conclusion

In conclusion, irrespective of differences between rPDL cells and hPDL cells, heterogeneous rPDL cell population demonstrated characteristic phenotype of MSCs, with cells that were clonogenic, proliferative and showing multi-differentiation potential, alike hPDL cells. Therefore, the null hypothesis was not rejected. In addition, instead of isolating a homogeneous population of rPDLSCs, heterogenous rPDL cells might be an option and could have the potential to be used for a multitude of applications in preclinical animal trials such as periodontal regeneration, and as a source of MSCs for translational research in regenerative medicine.
